# Periodontal Disease: The Good, The Bad, and The Unknown

**DOI:** 10.3389/fcimb.2021.766944

**Published:** 2021-12-07

**Authors:** Lea M. Sedghi, Margot Bacino, Yvonne Lorraine Kapila

**Affiliations:** ^1^ School of Dentistry, University of California, San Francisco, San Francisco, CA, United States; ^2^ Department of Oral and Craniofacial Sciences, School of Dentistry, University of California, San Francisco, San Francisco, CA, United States; ^3^ Department of Periodontology, School of Dentistry, University of California, San Francisco, San Francisco, CA, United States

**Keywords:** dysbiosis, host response, oral microbiome, periodontal pathobionts, oral-systemic association

## Abstract

Periodontal disease is classically characterized by progressive destruction of the soft and hard tissues of the periodontal complex, mediated by an interplay between dysbiotic microbial communities and aberrant immune responses within gingival and periodontal tissues. Putative periodontal pathogens are enriched as the resident oral microbiota becomes dysbiotic and inflammatory responses evoke tissue destruction, thus inducing an unremitting positive feedback loop of proteolysis, inflammation, and enrichment for periodontal pathogens. Keystone microbial pathogens and sustained gingival inflammation are critical to periodontal disease progression. However, recent studies have revealed the importance of previously unidentified microbes involved in disease progression, including various viruses, phages and bacterial species. Moreover, newly identified immunological and genetic mechanisms, as well as environmental host factors, including diet and lifestyle, have been discerned in recent years as further contributory factors in periodontitis. These factors have collectively expanded the established narrative of periodontal disease progression. In line with this, new ideologies related to maintaining periodontal health and treating existing disease have been explored, such as the application of oral probiotics, to limit and attenuate disease progression. The role of systemic host pathologies, such as autoimmune disorders and diabetes, in periodontal disease pathogenesis has been well noted. Recent studies have additionally identified the reciprocated importance of periodontal disease in potentiating systemic disease states at distal sites, such as in Alzheimer’s disease, inflammatory bowel diseases, and oral cancer, further highlighting the importance of the oral cavity in systemic health. Here we review long-standing knowledge of periodontal disease progression while integrating novel research concepts that have broadened our understanding of periodontal health and disease. Further, we delve into innovative hypotheses that may evolve to address significant gaps in the foundational knowledge of periodontal disease.

## The Oral Microbiome: Heroes and Villains

### ​​The Goodfellas

The oral cavity is home to approximately 700 species of bacteria that together comprise the oral microbiome (Deo and Deshmukh, 2019). The oral microbiome is composed of a unique and diverse ecosystem of microbial organisms that metabolically and physically interact. Such interactions result in the formation of complex biofilm communities in which physio-chemical gradients create distinct niches for microorganisms of differing metabolic needs (Rosan and Lamont, 2000; Kim et al., 2020). Work by Mark Welch et al. demonstrates the spatio-chemical structure of healthy supragingival plaque, described by a “hedgehog-like” structure organized in a radial fashion (Mark Welch et al., 2016). In this model, *Corynebacterium spp* anchor to early colonizers, such as *Actinomyces spp* and *Streptococcus spp*, and radially extend outward to provide a long, annulus structure. Attached at the tip of the annulus, *Haemophilus, Aggregatibacter*, and *Neisseriaceae* occupy the oxygen- and nutrient-rich periphery. Metabolic output from oxidative species at the periphery creates an anoxic environment at the biofilm center, in which anoxic capnophilic species, such as *Capnocytophaga, Leptotrichia*, and *Fusobacterium* thrive along the middle of the annulus. This study also found striking similarities in the composition of supragingival plaque and subgingival plaque in healthy subjects, identifying 13 genera with at least 3% abundance that constituted 85% of all sequencing in supragingival plaque and more than 80% of all subgingival plaque (Mark Welch et al., 2016). When the complex ecosystem of the oral biofilm is perturbed, microbial dysbiosis ensues (Hajishengallis and Lamont, 2012). This disruption in microbial community dynamics plays a major role in the etiology of gingivitis and development of periodontal disease. Periodontitis is also characterized by immune dysregulation and inflammation and increased representation of periodontal pathogens that bi-directionally promote one another and together drive destruction of the tooth supporting structures, including the periodontal ligament (PDL) and alveolar bone (Ebersole et al., 2013). The impact of chronic inflammatory diseases at sites far from the oral cavity on periodontitis, and the emerging role of periodontitis in systemic inflammation, is also becoming recognized in the pathogenesis of periodontal disease (Qasim et al., 2020).

### Breaking “Bad”

The polymicrobial and synergy model, proposed by Lamont and Hajishengallis (2013), integrates aspects of various proposals of periodontal disease etiology (Hajishengallis and Lamont, 2013). For example, the ecological plaque hypothesis proposed by Marsh (1994), the red complex discovery by Socransky et al. (1998), the model of synergistic interactions between keystone pathogens and commensals (Hajishengallis et al., 2011), and studies completed on the immune network that delineates states of health and disease (Dutzan et al., 2016) are collectively combined to grasp the complex nature of disease progression, including the role of synergistic microbial communities as well as keystone pathogens, and the role of oral immune network dysregulation. It is widely accepted that periodontitis is driven by many factors including host immunity, host environmental factors, and keystone periodontal pathogens that are critical to disease etiology. A comprehensive model of multi-species interactions in periodontitis was defined by Socransky et al. (1998) who, using genomic DNA probes and checkerboard DNA-DNA hybridization, identified *Porphyromonas gingivalis, Tannerella forsythia, and Treponema denticola* as species which appear together at higher frequency with increasing severity of periodontal disease, thus coining the cluster “the red complex” (Socransky et al., 1998; Holt and Ebersole, 2005). Over the years, however, genera associated with periodontitis have expanded beyond the red complex to include *Filifactor alocis*, *Porphyromonas*, *Synergistetes*, and *Peptostreptococcaceae (* Griffen et al., 2012; Abusleme et al., 2013), as well as *Actinomyces* a*ctinomycetemcomitans* that is associated with aggressive periodontitis (Slots et al., 1980; Haubek et al., 2008).

Many studies have investigated the abundance of certain phyla and genera that differentiate between periodontal health and disease. Although many discrepancies exist between studies, a shift in relative proportions of the four most abundant phyla, including Bacteroidetes, Actinobacteria, Proteobacteria and Firmicutes, and more specifically a decrease in the abundance of Proteobacteria and Actinobacteria and increased abundance of Bacteroidetes and Firmicutes in periodontitis, is unvarying amongst different investigators (Griffen et al., 2012; Wang et al., 2013; Cai et al., 2021). On a genus level, *Treponema* is overrepresented in periodontal disease (Abusleme et al., 2013; Cai et al., 2021), and conversely *Rothia, Actinomyces*, and *Neisseria* dominate communities of health (Abusleme et al., 2013; Rosier et al., 2020; Cai et al., 2021). As the understanding of the species involved in the etiology of periodontitis grows, the relationship between such pathogens, pathobionts, and oral symbionts among and across one another has revealed a complex network comprised of antagonistic and synergistic interactions among members of the oral microbiome. For example, pathobiont *Fusobacterium nucleatum* has been shown to increase the survivability of putative periodontal pathogen *P. gingivali*s in aerated conditions (Bradshaw et al., 1998). Pathogen-pathogen potentiation has been demonstrated by mutualistic interactions between *T. denticola* and *P. gingivalis*, in which *T. denticola* benefits from succinate produced as a metabolic byproduct by *P. gingivalis (* Grenier, 1992). Moreover, Tan et al. (2014) found that *T. denticola* and *P. gingivalis* densities increased significantly in co-culture compared to monocultures (Yamada et al., 2005; Ng et al., 2019). It was also found that these two species respond to each other in co-culture by altering the expression of many genes, including glutamate and glycine catabolism by *T. denticola* and shifts in fatty acid and thiamine pyrophosphate synthesis by *P. gingivalis (* Tan et al., 2014). Moreover, *A. actinomycetemcomitans* and *Filifactor alocis*, both associated with localized aggressive periodontitis, display mutualistic community growth (Wang et al., 2013). Dual-species interactions of periodontal pathogens in relation to disease severity was also demonstrated in a murine model in which co-infection by *T. forsythia* and *F. nucleatum* induced significantly increased alveolar bone loss compared to mono-species infected groups (Settem et al., 2012). Additionally, the role of symbiont-pathogen interactions to disease potentiation has been demonstrated by dual species infection with *Streptococcus gordonii* with *P. gingivalis* that was found to promote significantly greater bone loss compared to mono-species infection (Daep et al., 2011). In line with pathogen interactions with symbionts, *P. gingivalis* has been found to strongly associate with *Streptococcus oralis (* Maeda et al., 2004).

A study by Peterson et al. (2014) analyzing the cooperative and antagonistic relationships of the oral microbiome uncovered that most inter-species interactions are positively correlated. This positively associated network includes the genera *Bacteroides*, *Eubacterium*, *Filifactor*, and *Fusobacterium, Peptostreptococcus*, *Campylobacter*, *Johnsonella*, and *Parvimonas* (Peterson et al., 2014). This study supports other findings that have identified that shifts in the abundance of a single species is often accompanied by concurrent shifts among other species due to interspecies synergism and antagonism (Henne et al., 2014; Zhou et al., 2016; Abusleme et al., 2021). To uncover the function associated with specific transcripts, this study also analyzed the transcriptome of microbial samples and found that transcripts encoding ribosomal subunit biogenesis and carbohydrate utilization were most abundant (8.9% and 10%, respectively) among total samples. They also found that most stress response transcripts (50-75%) were associated with oxidative stress (Peterson et al., 2014). Although not focused specifically on the functional networks associated with periodontal disease, this study nonetheless highlights the importance of understanding not only the composition of the oral microbiome, but additionally the functional networks that exist in health and that may become perturbed in disease. More research must be completed on the synergistic relationships in health and disease and how such interactions collectively translate to shifts in community structure and disease emergence, progression, severity, and response to treatment (Takahashi, 2015).

### Structure Meets Function

Utilization of multi-omics approaches to address questions of both microbial community function and underlying mechanisms has further contributed to our understanding of microbial composition in health versus periodontitis and moreover has identified functional genes and microbial metabolic pathways that are over-represented in periodontal disease. Wang et al. (2013) identified significant differences among the four most abundant phyla, Bacteroidetes, Actinobacteria, Proteobacteria and Firmicutes, in periodontitis, with glycan biosynthesis and metabolism being over-represented and carbohydrate metabolism, amino acid metabolism, energy metabolism, lipid metabolism, membrane transport, and signal transduction being under-represented among diseased cohorts (Wang et al., 2013). Metagenomic analysis of collective data from nine peer-reviewed publications, in which a total of 943 subgingival samples from periodontitis-afflicted and healthy subjects identified enriched pathways exclusive to each cohort: significant differences (*p<0.05*) in cell motility, cellular processing and signaling, nucleotide metabolism, metabolism of cofactors and vitamins, and nervous system function were significantly different, with an increase in bacterial motility proteins and assembly among periodontitis patients. Additionally, a significant difference *(p<0.05)* was identified for the synthesis and degradation of ketone bodies, nitrogen metabolism, and sulfur metabolism (Cai et al., 2021).

In a multi-omics analysis of an experimental gingivitis model, a shift in microbial composition, metabolite production, and salivary cytokines at 24 to 72 hours following oral hygiene suspension was identified, thus suggesting a critical window in disease onset and progression. Results from the experimental gingivitis model were subsequently compared to raw data from previously published microbiome data sets. Profound similarities among microbial community shifts were identified among studies, with cohorts spanning from the United Kingdom, the United States, and China (Huang et al., 2021). A study by Yost et al. (2015) compared subgingival samples from individuals with both active and stable periodontitis to a previous study on periodontal signatures. Functional signatures were identified among microbial communities defining states of disease *versus* health, with upregulation of red complex genes associated with transport, proteolysis, protein kinase C-activating G-protein coupled receptor signaling pathway and response to antibiotic and downregulation of genes associated with cobalamin (vitamin B12) biosynthesis pronounced among the diseased group. Importantly, this study determined that various species not explicitly defined as putative periodontal pathogens, including *Streptococcus mitis, Streptococcus intermedius*, and *Veillonella parvula* additionally contributed to disease progression. Such findings highlight the role of microbial community compositional and functional dysbiosis in driving periodontal disease pathogenesis (Yost et al., 2015).

### Antibiotics

The nature of periodontists as a complex polymicrobial biofilm in dysbiosis has led to difficulty in treatment. Compounding, the oral microbiome is a significant reservoir of mobile antibiotic resistant genes than can be transferred to pathogenic microbes (Roberts and Mullany, 2010; Carr et al., 2020). A study by Rams et al. found that 74.2% of patients (n=400 adults with chronic periodontitis) had periodontal pathogens resistant to at least one antibiotic tested doxycycline, amoxicillin, metronidazole, or clindamycin (Rams et al., 2013). Sgolastra et al. (2021) completed a systematic review of 21 randomized clinical trials of chronic periodontitis and found that scaling and root planning with the addition of amoxicillin (AMX) + metronidazole (MTZ), as compared to scaling and root planning alone, reached the highest probing depth reduction at 6 and 12 months, and clinical attachment gain at 6 and 12-months (Sgolastra et al., 2021). Although such treatments have proved more successful, the systemic effects of broad-spectrum antibiotic use must be considered as well with rising incidences of antimicrobial resistance. Growing research has linked perturbations in the microbiome with antibiotic use (Blaser, 2016; Faber et al., 2016; Mohajeri et al., 2018; Maier et al., 2021). The World Health Organization (WHO) has deemed antimicrobial resistance as one of the top ten global health threats facing humanity (World Health Organization, 2014). Under these considerations, innovative targeted approaches must be implemented in combatting periodontitis. One such treatment is the novel antibiotic, amixicile, which inhibits anaerobic bacteria implicated in periodontitis. including *P. gingivalis, T. forsythia, T. denticola*, and *F. nucleatum* through inhibition of pyruvate ferredoxin oxidoreductase (PFOR) (Hutcherson et al., 2017; Gui et al., 2020). Amixicile targets the cofactor of PFOR rather than the enzyme itself, which reduces the risk of antibiotic resistance by mutation (Hutcherson et al., 2017). Additionally, amixicile has been found to suppress the growth of oral anerobic bacteria while leaving aerotolerant bacteria unaffected (Hutcherson et al., 2017; Gui et al., 2020). A multifaceted approach incorporating the use of targeted antimicrobials with treatments that support the rehabilitation of a healthy oral microbiome may lead to improved health outcomes of patients with periodontitis.

### The New Kids in Town: The Oral Virome in Periodontal Disease

The role of the oral microbiota in periodontal disease progression has largely focused on dysbiosis related to bacterial species. However, the role for the oral virome, composed of bacteriophages, viruses, and retroviruses, among the oral microbiota in periodontitis remains largely limited. Viruses have been recognized as constituents of the oral microbiota in health and disease (Ly et al., 2014) and certain viruses, such as Epstein-Barr virus, herpes simplex virus, and cytomegalovirus have been implicated in a variety of oral pathologies (Bilder et al., 2013; Kato et al., 2013; Contreras et al., 2014; Slots, 2015; Zhu et al., 2015; Lu et al., 2016; Gao et al., 2017; Naqvi et al., 2018; Slots, 2019). Findings related to the role of viruses in periodontal disease etiology, including disease potentiation by viruses *via* interaction with periodontal pathogens, viral infection of host cells, and viral-mediated biofilm dysbiosis, were comprehensively outlined by Martínez et al. (2021) Comparatively, however, the significance of the oral virome in periodontitis has not been fully elucidated. A polymicrobial infection model of periodontal disease composed of an inoculum containing *P. gingivalis, F. nucleatum, T. denticola*, and *T. forsythia* was employed in a murine model over the course of 8 weeks to understand the effects of infection on PDL properties, alveolar bone loss, the host serum immune profile, and the resident oral microbiota (Gao et al., 2020). Oral swabs were collected prior to administering the infection (following antibiotic treatment) and at 1, 4, and 8 weeks post-infection to understand longitudinal changes to the oral microbiota in parallel with disease progression over time. Maxillary and mandibular specimens were utilized to perform metagenomic shotgun sequencing to examine perturbations to the oral microbiota in response to the pathogenic inoculum. Bacterial community composition and diversity did not differ significantly between control and infection groups (*p=0.92).* However, significant changes to the oral virome were detected between infection and control groups *(p=0.04).* Viral members associated with increased bone loss among the infection group included Gammaretrovirus, Porcine type-C oncovirus, Bat Gammetrovirus, and Golden hamster intracisternal A particle H18. Porcine type-c oncovirus was additionally associated with increased PDL space. With regards to the immune response, a weak association was found between Gibbon ape leukemia virus and immune gene *Tnfsf 14* (Gao et al., 2020). *A* significant role for the virome has also been recognized in other chronic disease states, some of which are also associated with periodontitis, including inflammatory bowel disease, diabetes, and cancer (Santiago-Rodriguez and Hollister, 2019).

**Figure 1:**
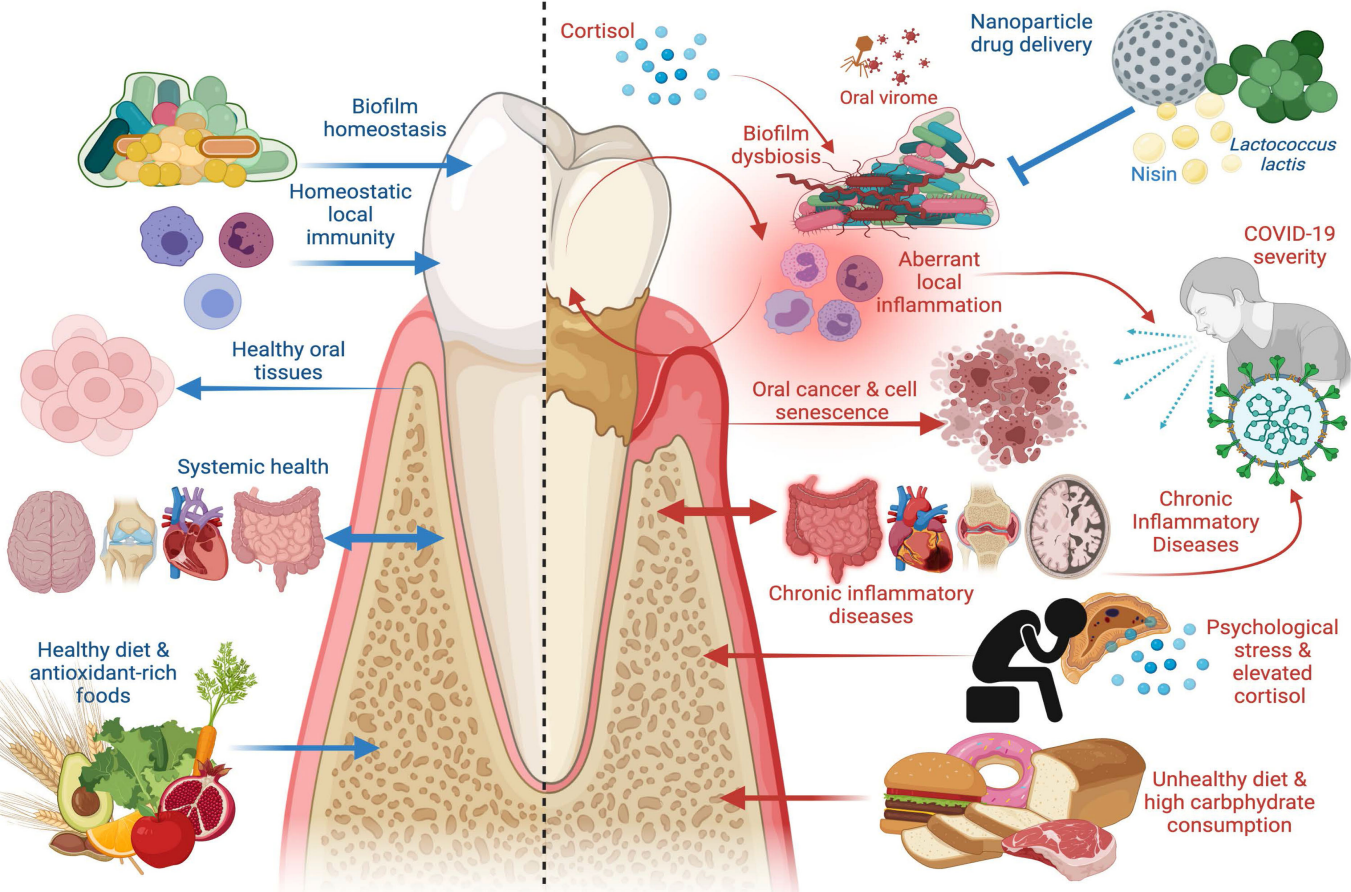
The Good and the Bad in Periodontal Disease. Left panel: factors that promote periodontal health, including supra- and subgingival biofilm homeostasis, homeostatic immunity in gingival and periodontal tissues, healthy dietary constituents, and absence of chronic inflammatory disease at distant sites. Healthy periodontal tissues in turn reduce risk of oral carcinogenesis and bi-directionally affect systemic health such that chronic inflammatory disease risk is reduced. Right panel: factors that promote periodontal disease, including biofilm dysbiosis, uncontrolled gingival and periodontal inflammatory responses, psychological stress paralleled by elevated cortisol release, and unhealthy diets characterized by high carbohydrate consumption. Periodontal disease adversely affects oral tissue health and is a risk factor in oral carcinoma, induces cell senescence in healthy cells, promotes systemic inflammation, and is a risk factor in chronic inflammatory diseases including inflammatory bowel disease (IBD), cardiovascular disease, autoimmune conditions, and Alzheimer’s disease. Periodontal disease has emerged as a risk factor in COVID-19 severity and poor outcomes, as are other systemic chronic inflammatory diseases. New treatment modalities, such as oral probiotics including *L. lactis* and bacteriocins, such as nisin, that can be paired with nanoparticle drug delivery systems, have emerged as potential therapeutics to re-establish biofilm homeostasis and modulate aberrant inflammation. Image created with www.Biorender.com.

Most viruses present in the oral cavity are bacteriophages (Pride et al., 2012; Robles-Sikisaka et al., 2013), many of which belong to the Caudovirus families, Siphoviridae, Myoviridae, and Podoviridae (Wichels et al., 1998; Sullivan et al., 2003). Like bacterial constituents of the oral microbiome, the virome is altered by environmental influences and is highly variable amongst individuals (Pride et al., 2012; Robles-Sikisaka et al., 2013; Abeles et al., 2014). Moreover, oral viruses have been shown to elicit host immune responses, thus implicating a role for periodontal disease pathogenesis in the crosstalk between host immunity and the oral microbiota (**Figure 1**) (Duerkop and Hooper, 2013; Abeles et al., 2014). A study by Ly et al. (2014) sought to define differentiating characteristics of the oral virome in health *versus* periodontitis. Saliva and oral biofilm samples were collected from 16 subjects that were periodontally healthy or had mild to significant periodontitis. Salivary viromes largely clustered according to periodontal disease status. This was also reflected among supra- and subgingival biofilm samples in which viromes from subjects with significant periodontitis clustered together. The proportion of shared virome homologues was greater among subjects with severe periodontal disease (*p=0.002)* compared to subjects with mild periodontitis or healthy status for subgingival plaque samples. Significant differences in periodontally healthy subjects compared to diseased subjects was also identified in relationship to oral biogeographical site. In healthy subjects, *Siphviridae* were identified as the most abundant viral family. The abundance of podoviruses from each intraoral site among healthy subjects was also similar, however the relative abundance of myoviruses varied considerably with biogeographical location and disease state. While myoviruses were significantly more abundant in saliva from healthy individuals compared to diseased cohorts, they also were more abundant among subgingival plaque from the periodontitis cohorts compared to healthy subjects. No significant differences in viral families were observed in supragingival plaque, however. Together, these findings suggest that the oral virome is significantly altered in subgingival plaque due to increased myovirus abundance. At a higher taxonomic level, viruses belonging to *Firmicutes* and *Proteobacteria* were most abundant in saliva, followed by *Bacteroidetes* and *Actinobacteria.* Conversely, those associated with *Proteobacteria* and *Bacteroidetes* were most abundant in biofilm samples. Bacteriophage, the most abundant viruses observed in the oral cavity, serve as significant drivers of bacterial diversity in varying microbial ecosystems, and as such may manipulate bacterially mediated aspects of periodontitis (Willner et al., 2009; Reyes et al., 2010; Minot et al., 2011; Foulongne et al., 2012; Pride et al., 2012; Minot et al., 2013; Ly et al., 2014). Thus, it is important to better understand the oral virome for both its potential direct aspects to periodontal disease and its possible role in bacterially mediated disease potentiation.

## Better Safe Than Sorry? The Necessities and Pitfalls of Periodontal Immunity

Continued accumulation of supra- and subgingival polymicrobial biofilm communities evokes a persistent host immune response within the periodontium (Hajishengallis et al., 2020). This inflammatory process can be reversed if microbial biofilm is removed, and the inflammation is limited to involvement of the gingival epithelium and connective tissues. However, the inflammatory process becomes irreversible if biofilm accumulation persists and leads to involvement of deeper periodontal tissues, such as deepening of the gingival crevice, destruction of the PDL, and alveolar bone loss, at which point the disease progresses from gingivitis to periodontitis (Armitage, 2004). This sustained inflammation is further encouraged by the resulting outgrowth of periodontal pathogens that thrive under inflammatory conditions, during which proteinaceous by-products of tissue destruction (i.e., collagen breakdown products, amino acids, iron, heme, etc.) reinforce pathogen outgrowth (Hajishengallis, 2014; Herrero et al., 2018; Rosier et al., 2018). Periodontal disease severity is defined by increasing complexity of inflammatory cell infiltrate. The Page and Schroeder model describes four distinct stages characterizing disease progression: the initial lesion being dominated by neutrophils, the early lesion characterized by elevated macrophages and T cells, and the later established and advanced lesions being characterized by B cell and plasma cell involvement (Page and Schroeder, 1976). Although microbial challenge is necessary for disease onset, host inflammatory involvement is the primary driving force for periodontal tissue destruction (Hasturk and Kantarci, 2015; Hajishengallis and Korostoff, 2017).

### It Takes Two to Tango: Microbial-Host Inflammatory Processes

The presence of pathogens in the gingival pocket elicits chemokine secretion by epithelial cells that recruit neutrophils from systemic circulation to the junctional epithelium (Bosshardt and Lang, 2005; Fujita et al., 2018). Protective proteolytic responses by neutrophils consequently disrupts the epithelium, thereby promoting pathogen invasion deeper into epithelial tissues and into the lamina propria, enhancing tissue breakdown and bone resorption (Bosshardt and Lang, 2005). Periodontal pathogens have evolved abilities to hijack and manipulate the host inflammatory response to promote inflammation while contrarily evading such responses. Bacteria affect epithelial barrier function *via* directly manipulating host genes and/or proteins involved in barrier function as well as *via* indirect mechanisms involving immunoregulatory responses (Turner, 2009). When homeostatic communication between the oral microbiome and host immune response is perturbed, either *via* microbial or inflammatory stimuli, dysbiosis is perpetuated and the deleterious effects of periodontitis are cyclically perpetuated. Tissue destruction in periodontitis is driven by an expansion of T_H_17 cells in an interleukin (IL)-6 and IL-23 dependent manner, due to changes in microbial community structure (Dutzan et al., 2018).

Deleterious microbial community remodeling is largely attributed to increased representation and integration of the keystone periodontal pathogen, *P. gingivalis*, which drives increased bacterial load, changes in the composition of the microbiota, and induces significant bone loss in specific pathogen free (SPF) mice. Interestingly, although found to increase bacterial load, *P. gingivalis* only comprised 0.01% of the total bacterial count. Moreover, *P. gingivalis* is not capable of driving bone loss in germ free (GF) mice, thus suggesting that, by itself, it does not solely contribute to periodontal destruction (Hajishengallis et al., 2011). *P. gingivalis* also directs important inflammatory perturbances observed in periodontal disease, such as modulating cross talk between toll-like receptor (TLR)-2 and C5aR. Such interactions disrupt the TLR-MyD88 pathway and instigate the proinflammatory TLR2-PI3K signaling pathway, which causes inflammation through reduction in phagocytosis and by enhancing intracellular survival of *P. gingivalis (* Maekawa et al., 2014; Makkawi et al., 2017).

### Revved *Up*: Microbial-Host Tissue Destructive Networks and Profiles

In periodontitis, breakdown of the extracellular matrix and alteration of the periodontal ligament space is observed (Armitage, 2004). Recently, Malone et al. characterized a mechanism by which *T. denticola* mediates direct effects to cell barrier function *via* actin remodeling dynamics in periodontal ligament cells (Malone et al., 2021). Immunofluorescence staining revealed that challenge with *T. denticola* reduced actin stress fiber abundance, complemented *via* a 30% decrease in β-actin protein expression observed by Western blotting. RNA-sequencing corroborated such findings such that, upon challenge with *T. denticola*, PDL cells demonstrated upregulation of actin and cytoskeletal-related pathways, including Ras protein signal transduction and regulation of small GTPase-mediated signal transduction. From these genes, *RASA4* was identified as significantly upregulated upon *T. denticola* challenge. The role of *T. denticola’s* effector protein dentilisin in actin reorganization was next investigated, in which purified dentilisin was sufficient to enhance RASA4 upregulation. Matrix metalloproteinase (MMP)-2 activation is increased upon challenge with *T. denticola*; as such, the effect of *T. denticola*-mediated changes in actin dynamics and the effect of this on MMP-2 activity in PDL cells was investigated. *T. denticola* significantly increased MMP-2 activity, and this effect was abrogated by polymerizing agent Jasplakinolide and increased by de-polymerizing agent Latrunculin B, indicating that *T. denticola* promotes MMP-2 expression *via* actin depolymerization (Malone et al., 2021).

Periodontal pathogens additionally promote tissue destruction by up-regulating tissue-destructive genes in host tissue. Periodontal ligament fibroblasts undergo tissue remodeling *via* expression of hydrolytic enzymes including MMPs and additionally contribute to production of pro-inflammatory mediators such as cytokines and chemokines. Moreover, PDL cells modulate expression of pattern recognition receptors (PRR) and TLRs that contribute to surveying the environment for microbial species in the periodontium (Sun et al., 2010; Tang et al., 2011; Jönsson et al., 2011; Sokos et al., 2015; Zhang et al., 2015; Jiang et al., 2016). Unresolved TLR signaling leads to overactivation of genes involved in tissue destruction, such as those encoding MMPs (Li et al., 2011; Sapna et al., 2014; Sokos et al., 2015). Lipoproteins are recognized as significant virulence factors associated with TLR2 stimulatory bacterial ligands (Sela et al., 1997; Wilson and Bernstein, 2016; Sobocińska et al., 2018). Elevated *T. denticola* levels among periodontitis patients are complemented by increased MMP levels in periodontal tissues (Loesche, 1988; Ramseier et al., 2009; Ateia et al., 2018). Ganther et al. recently investigated *T. denticola*-mediated MMP expression *via* its effector protein, dentilisin. PDL cells challenged with *T. denticola* demonstrated increased expression of genes involved in extra-cellular matrix (ECM)-receptor, collagen degradation, and degradation of the ECM among the 20 significantly enriched biological processes (Ganther et al., 2021). While not significant, MMP-2 and MMP-14 were upregulated upon *T. denticola* challenges, corroborated by prior research demonstrating their involvement in periodontal disease (Lint and Libert, 2007; Page-McCaw et al., 2007; Ateia et al., 2018). Upregulation of MMP-2 and MMP-14 was specific to *T. denticola*, as challenge with the Gram-negative commensal *Veillonella parvula* did not result in upregulation of these MMPs. The role of effector protein dentilisin on MMP regulation was next investigated, in which challenge with purified dentilisin resulted in upregulation of MMPs. Conversely, challenge with dentilisin-deficient *T. denticola* did not induce MMP activation. To establish a direct association of TLR2 activation to MMP gene regulation in PDL cells, shRNA knockdown of *TLR2* was performed and cells were challenged with purified dentilisin or *T. denticola.* Upon treatment with either purified dentilisin or *T. denticola, MMP-2, -11, -14, -17*, and -*28* expression were significantly increased. The role of Myd88 in TLR2 activation was next investigated, as most TLRs signal through MyD88 (Wang et al., 2017). shRNA was used to knockdown *MyD88.* Knockdown lines treated with *T. denticola* or purified dentilisin did not induce upregulation of MMP targets compared to control samples. Specificity factor protein-1 (Sp-1) is a target of TLR/MyD88 associated with tissue destruction and pro-inflammation. To determine if Sp1 expression is altered by *T. denticola* in PDL cells, cells were challenged with wild type *T. denticola* or control dentilisin mutant *T. denticola*-CF522 followed by western blot analysis using a monoclonal antibody against Sp1. While treatment with wild-type cells increased Sp1 expression, challenge with *T. denticola*-CF522 failed to do so. Collectively, this study linked dentilisin with TLR2 activation and identified potential tissue specific inducible MMPs that may play additional roles in mediating host inflammatory responses in periodontal disease (Ganther et al., 2021).

### The Innocent Bystanders: Cell Senescence

The underlying mechanisms of microbial dysbiosis and host inflammatory responses continues to develop, contributing to a greater understanding of these processes, and alternative mechanisms by which periodontal tissue destruction occurs continue to be revealed. Recruited neutrophils secrete pro-inflammatory cytokines and reactive oxygen species (ROS) to eliminate pathogens from affected tissues. Host immunity paradoxically perpetuates periodontal disease severity *via* further promoting microbial dysbiosis (Ebersole et al., 2013; Cekici et al., 2014). In line with this concept, emerging evidence suggests that chronic inflammation in the periodontium further promotes inflammatory processes and tissue destruction *via* cellular senescence among healthy resident cells that are chronically exposed to an inflammatory environment (Aquino-Martinez et al., 2020). Although ROS can protect against invading bacteria, they can also cause harm to healthy host cells, thus inducing stress-mediated DNA damage (Barzilai and Yamamoto, 2004). ROS generated *via* inflammatory processes can damage cellular DNA of resident periodontal cells. Additionally, exposure to bacterial lipopolysaccharide (LPS) can also promote DNA damage among gingival and alveolar bone cells (Cheng et al., 2015; Aquino-Martinez et al., 2020). Damaged DNA can undergo repair; however, chronic damage to the genome elicits an apoptotic or senescent response upon exposed cells (Childs et al., 2014). Cells that acquire a senescent phenotype overexpress pro-inflammatory cytokines, including but not limited to IL-6, IL-1β, and IL-8, and proteolytic enzymes including MMP-1, MMP-3, MMP-12, and MMP-13 (Coppé et al., 2010; Aquino-Martinez et al., 2020; Levi et al., 2020). Such cells are referred to as having a senescence-associated secretory phenotype (Coppé et al., 2010). Secretion of pro-inflammatory signals, proteolytic enzymes, and ROS alters the periodontal environment, perpetuating inflammatory cell infiltration and tissue damage that are hallmarks of periodontal disease. Senescent cells may further contribute to the inflammatory positive feedback loop in periodontitis in that DNA damage and chronic inflammation can drive senescence-induced inflammatory processes, and chronic inflammation thereby facilitates generation of ROS and stress-mediated DNA damage (Jurk et al., 2014; Mittal et al., 2014).

Four primary mechanisms may contribute to senescence related to periodontal disease progression: persistent insult by Gram-negative pathogens, chronic inflammation, continued repair of damaged tissues, and bacteria-induced immunosuppression. Persistent exposure to LPS from Gram-negative pathogens induces a genotoxic effect among gingival fibroblasts (Cheng et al., 2015). Continued exposure to LPS of *P. gingivalis* was shown to cause premature DNA damage-driven senescence within alveolar bone cells (Aquino-Martinez et al., 2020). Additionally, cytolethal distending toxin secreted by *A. actinomycetemcomitans* induces double-stranded breaks in human gingival epithelial cells, leading to apoptosis or cellular senescence (Guerra et al., 2011; DiRienzo, 2014; Grasso and Frisan, 2015). Chronic inflammation damages cellular DNA *via* continued generation of ROS and oxidative stress (Martindale and Holbrook, 2002; Van Houten et al., 2018). ROS production in gingival fibroblasts due to LPS exposure induced DNA damage, as demonstrated by increased expression of anti- and pro-apoptotic proteins. Senescent cells are also identified among repaired and/or regenerated tissues (Coppé et al., 2010; Campisi, 2013). The continued repair and renewal capacity of the gingival epithelium, albeit acting as a cellular-defense mechanism in response to mechanical damage and bacterial insult, may also contribute to periodontal tissue destruction (Bulut et al., 2006; Ji et al., 2015). In support of this hypothesis, rapamycin, an mTOR pathway inhibitor, delayed the onset of senescence characteristics and preserved the mitotic potential of healthy gingival fibroblasts (Xia et al., 2017). Senescent cells are also targeted by the host immune system to detect and remove damaged cells. However, the immune-suppressive capacities of various periodontal pathogens, including *P. gingivalis, A. actinomycetemcomitans*, and *T. forsythia* may promote accumulation of senescent cells. Findings regarding the role of cell senescence in periodontal disease progression may help to better understand the role of chronic inflammation in periodontitis, opening new avenues for disease prevention strategies *via* senotherapeutic treatment modalities (Aquino-Martinez et al., 2020).

### Holding Down the Fort: Homeostatic Gingival Barrier Defenses

Albeit the role of host inflammatory responses in the gingiva in disease progression, barrier immunity in the gingiva is critical as this site is prone to insult by regular masticatory challenges, microbial involvement, as well as dietary and airborne antigens (Dutzan et al., 2016). Such tissue-specific cues at the gingiva result in unique immune responses within periodontal tissues compared to other physiological barrier sites. The oral epithelial barrier that lines the interior of the gingiva is particularly vulnerable to bacterial insult. The wall of the gingival sulcus is lined with non-keratinized crevicular epithelium that progressively thins towards the base of the sulcus, at which point the mucosa meets the enamel surface of the tooth. At this point of interaction, the epithelium transitions to the junctional epithelium that is especially vulnerable, as it tapers to just 1-2 cell layers of thickness. Here the epithelium is attached to dental surfaces *via* hemidesmosomes, and this connection is highly permeable (Dutzan et al., 2016). At this site, gingival crevicular fluid flows and contains host immunological components, including plasma proteins, cytokines, immunoglobulins, and various immune cells (Buclkacz and Carranza, 2012; Lamster, 1997). The junctional epithelium, however, maintains the ability to regenerate in response to damage (Nanci, 2017), and such regenerative capabilities reflect that this barrier site is uniquely tailored to withstand a variety of bacterial, inflammatory, and mechanical stimuli (Nanci, 2017).

Microbiome-dependent and -independent mechanisms of immune homeostasis at the gingiva have been determined, with microbe-independent mechanisms holding a dominant role in homeostatic immunity (Dutzan et al., 2017). *Via* the junctional epithelium, neutrophils transmigrate into the periodontium. Neutrophils comprise most immune cells represented at the gingival crevice in health, constituting 95% of leukocytes (Dutzan et al., 2016; Rijkschroeff et al., 2016). The presence of neutrophils in germ-free mice demonstrates a microbiota-independent role for neutrophil surveillance in the gingiva. Neutrophils are additionally crucial to resolution of immune responses *via* downregulation of IL-23 in response to microbial-mediated inflammation (Serhan et al., 2008; Moutsopoulos et al., 2014; Ley, 2017; Moutsopoulos et al., 2017). Malfunctions in neutrophil responses to such insults, as demonstrated *via* various single-gene mutations that affect granulopoiesis, neutrophil recruitment and extravasation that result in severe periodontitis phenotypes, emphasize the importance of neutrophils to maintaining periodontal health. However, increased neutrophil representation in response to inflammation is a hallmark of reciprocal reinforcement of aberrant inflammation in periodontitis. As such, a delicate balance of neutrophils is critical to periodontal immunity (Kantarci et al., 2003; Eskan et al., 2012; Billings et al., 2017). Dendritic cells, macrophages, and monocytes also comprise the gingival immune network in health (Hovav, 2014; Dutzan et al., 2016). Such immune cells are indicated in preserving barrier integrity and immune regulation in the gingiva in response to bacterial insult (Steinmetz et al., 2016). Various dendritic cell populations reside in gingival tissues and increase during inflammation (Jotwani et al., 2001; Jotwani and Cutler, 2003). T cells, B cells, and innate lymphoid cells are also present in healthy gingival tissues. While the role of innate lymphoid cells and B cells in health remains to be fully elucidated, substantial attention has been attributed to the role of T cell populations in the gingiva (Dutzan et al., 2016; Dutzan and Abusleme, 2019).

T cell populations are crucial to periodontal health and periodontal disease pathology. In health, CD4^+^ T cells with memory phenotypes dominate gingival tissues (Dutzan et al., 2016). Both CD4^+^ and CD8^+^ memory T cells produce IL-1, IL-17, and IFN-γ cytokines. The role of T_H_17 cells in microbiome-independent mechanisms of host immune homeostasis has gained increasing attention. Dutzan et al. found that T_H_17 cells increase representation in gingival tissue with increasing age in a microbiome-independent manner (Dutzan et al., 2017; Dutzan et al., 2018). This contrasts with other barrier sites, such as the intestinal epithelial lining and skin, at which T_H_17 cell expansion relies on microbial presence (Ivanov et al., 2009; Naik et al., 2012). Mechanical damage from masticatory forces resulted in T_H_17 expansion in gingival tissues, thus demonstrating distinct immune responses in gingival tissues to this unique tissue-specific cue (Dutzan et al., 2017). Masticatory challenge additionally prompted innate barrier defenses in an IL-6-dependent manner (Dutzan et al., 2017; Dutzan et al., 2018). T_H_17 cells thus demonstrate a protective role in response to microbiome-independent stimuli, such as mechanical damage that manifests at the gingiva. Challenging this, IL-17^+^ T_H_17 cell expansion is a hallmark of periodontitis and health alike in response to microbial and mechanical stimuli, respectively (Dutzan and Abusleme, 2019; Takahashi et al., 2019). Distinct cellular sources of IL-17 were delineated in periodontal health *versus* periodontal disease: under homeostatic conditions, TCRγδ^+^ T cells are the major source of IL-17 and T_H_17 comprise the dominant IL-17^+^ population in disease. In contrast to microbiome-independent mechanisms observed in health, T_H_17 expansion in experimental periodontitis was dependent on microbial dysbiosis, as well as IL-6 and IL-23. Interestingly, 16S rRNA sequencing did not reveal the role for specific bacterial candidates in driving dysbiosis, as T_H_17 expansion occurred in response to overall changes in the microbiome induced by various antibiotic regimens targeting specific bacterial populations (Dutzan et al., 2018).

## It’s a Two-Way Street: The Roles of Oral Health in Systemic Health, and Vice Versa

Increased incidence of periodontal disease and disease severity have been linked to various pathologies at distant sites from the oral cavity. Systemic disease states related to periodontal disease that have achieved greatest recognition to date include cardiovascular diseases, rheumatoid arthritis, and diabetes (Hajishengallis and Chavakis, 2021). The oral manifestation of various systemic pathologies, such as in the case of Crohn’s disease, has also been recognized (Pittock et al., 2001). However, a growing appreciation for the role of oral health in exacerbating, and in some cases even driving, systemic pathologies has evolved within recent years. Additional pathologies at sites distant from the oral cavity, such as Alzheimer’s disease, have attained sizable recognition for their association with periodontal health. Such findings have evoked a wider appreciation for the association of oral health to systemic health. Here, we discuss further research that demonstrates newly recognized associations of periodontal disease to additional systemic conditions and the role of periodontitis in potentiating various pathological states from sites far from the oral cavity.

### Alzheimer’s Disease and Cognitive Dysfunction

Alzheimer’s disease is characterized by progressing neuro-degeneration that spans from mild cognitive impairment, memory loss, language and communication disorders, and psychological and behavioral disorders (Qiu et al., 2009). Patients suffering from Alzheimer’s often present with compromised periodontal health (Martande et al., 2014; Aragón et al., 2018; D’Alessandro et al., 2018), ostensibly induced by declined self-care and a neglect for oral health by caregivers. However, an increased recognition for the potential influence of periodontal disease as a contributory mediator in Alzheimer’s is increasingly recognized, such as *via* an increase in pro-inflammatory mediators, such as C-reactive protein (CRP), IL-6, IL-1β, and TNF-α, that may contribute to synapoptoxic β-amyloid and neurofibrillary fiber tangle deposition (Gaur and Agnihotri, 2015). A meta-analysis by Hu et al. (2021) comprehensively reviewed studies spanning across the last decade related to the association of periodontal disease as a contributing factor in Alzheimer’s disease development. From 162 publications, 13 studies matched the criteria for the meta-analysis. Pooled results from eight of the included studies determined that patients with periodontal disease had a significantly greater risk of developing Alzheimer’s compared to healthy patient cohorts (Cestari et al., 2016; Chen et al., 2017; Choi et al., 2019; Tiisanoja et al., 2019; de Oliveira Araújo et al., 2021). Moreover, periodontal disease was additionally associated with development of mild cognitive impairment, a transitional stage between normal cognition and dementia and often observed as a central early clinical manifestation of Alzheimer’s disease. Although no significant association was found for mild/moderate periodontitis and increased risk for Alzheimer’s, severe periodontal disease was significantly associated.

Three primary mechanisms by which periodontal disease may contribute to Alzheimer’s disease have been identified as follows: 1) increased peripheral pro-inflammatory cytokines that systemically affect the brain *via* neural, humoral, and cellular mechanisms (Schmidt et al., 2002; Holmes et al., 2003; Engelhart et al., 2004), 2) ectopic migration of periodontal bacteria and related molecules directly to the brain *via* blood and/or cranial nerves, and 3) leptomeninges that may act as a mode of communication between periodontal pathogens and microglia in the brain (Hashioka et al., 2019). Ectopic migration of periodontal pathogens to brain tissue has been confirmed by the presence of *P. gingivalis* and related gingipains, as well as *Treponema spp* in autopsy specimens from patients with Alzheimer’s disease (Riviere et al., 2002). Ectopic migration of periodontal pathogens to brain tissue was also observed in mice upon oral *P. gingivalis* infection that demonstrated brain pathogen infiltration as well as increased amyloid β1-42 levels that are known to comprise amyloid plaques observed in Alzheimer’s disease. Gingipains have the capacity to cleave tau proteins, suggesting a role for *P. gingivalis* in tau phosphorylation and consequent accumulation of insoluble tau forms observed in Alzheimer’s pathology (Konig et al., 2016; Dominy et al., 2019). In support of this, gingipain load correlated with tau protein and ubiquitin load in autopsy specimens. Additionally, mice treated with gingipain-deficient *P. gingivalis* did not develop increased amyloid β1-42 compared to groups treated with wild type *P. gingivalis*. Additional oral-derived bacteria have also been implicated in Alzheimer’s disease, including *Campylobacter rectus* and *Prevotella melaninogenica (* Beydoun et al., 2020). Moreover, in a mouse model of periodontitis that omitted infection, increased levels of insoluble β-amyloids and increased neuroinflammation was still observed, further supporting role for additional orally derived bacteria in promoting Alzheimer’s disease (Kantarci et al., 2018; Kantarci et al., 2020; Hajishengallis and Chavakis, 2021).

Most studies to date have characterized the association of periodontal disease and Alzheimer’s risk solely among elderly populations. However, the impact of periodontal disease status on Alzheimer’s disease risk among younger populations is also a significant concern, as Alzheimer’s disease pathology often primarily manifests in younger populations as mild cognitive decline. Additionally, the association between Alzheimer’s and periodontal disease can more affirmatively be established among younger populations with fewer opportunities for comorbidities that may confound study findings. Most importantly, identifying periodontal disease as a significant risk factor for Alzheimer’s disease development may allot additional detection and interventional opportunities for Alzheimer’s disease (Hategan et al., 2021). Episodic memory among younger individuals (<45 y/o) among 60 subjects with either chronic, aggressive, or no periodontal disease status was assessed by Hategan et al. (2021) *via* delayed recall and immediate memory, as tested by the Rey Auditory Verbal Learning Test (RAVLT). The Montreal Cognitive Assessment test (MOCA) and Mini-Mental state Examination (MMSE) and Prague tests were also used (Lam et al., 2013; Hategan et al., 2021). Delayed and immediate recall scores were significantly lower among subjects with periodontitis, in which a significant difference in RAVLT recall scores was identified among the periodontal disease groups. Moreover, RAVLT delayed and immediate recall scores were also lower among the aggressive periodontitis group compared to the chronic and healthy cohorts. MOCA recall scores were also consistent with this. Salivary IL-1β and TNF-α were also assessed as pro-inflammatory molecules related to periodontitis. While TNF-α levels did not demonstrate significant association to any cognitive tests, IL-1β levels were significantly associated with Rey immediate recall ability (Hategan et al., 2021). This study ultimately demonstrated that young, healthy subjects without periodontal disease had improved episodic memory and learning rate compared to young, healthy subjects with aggressive periodontal disease.

### Systemic Lupus Erythematosus

Systemic lupus erythematosus (SLE) and periodontal disease are characterized by shared causative factors, spanning from those environmental, genetic, immunological, and microbiological in nature. Moreover, oral manifestations of SLE, including but not limited to mucosal ulcerations, xerostomia, hyposalivation, discoid lesions, cheilitis, and erythematous patches have additionally been noted (Khatibi et al., 2012; Benli et al., 2021), further supporting a plausible link between SLE and periodontal disease status. Furthermore, the prevalence of periodontitis in SLE patients is substantial, ranging from 60% to 94% of all SLE patients (Calderaro et al., 2016), and SLE patients additionally exhibit greater periodontal disease severity compared to non-SLE individuals with periodontitis, with greater clinical attachment loss and increased probing pocket depth (Wang et al., 2015; Corrêa et al., 2017; Zhang et al., 2017). Oral dysbiosis in periodontitis may trigger aberrant inflammatory responses observed in SLE. Conversely, the genetic and environmental risk factors in SLE may contribute to the initiation or maintenance of sustained inflammation in periodontal tissues. Various studies have identified mechanisms by which SLE may potentiate periodontal disease pathogenesis *via* immune dysregulation, tissue destruction, and alteration of the subgingival microbiota. SLE-induced inflammatory changes to the periodontium may promote a dysbiotic subgingival microbiota. In support, inflammatory cytokines IL-6, IL-17A, and IL-33 were increased in the saliva of SLE/periodontal disease patients compared to non-SLE subjects with periodontal disease (Bunte and Beikler, 2019). Interestingly, SLE patients also demonstrated increased serum antibodies against periodontal disease-associated oral bacteria such as *A. actinomycetemcomitans, P. gingivalis*, and *T. denticola.* Genetic ties between periodontal disease and SLE also exist; genetic variants associated with SLE, such as Fcy receptor genotypes PIIA, PIIIA and PIIIb, are higher among patients with co-SLE and periodontal disease pathologies. This finding supports that the connection between SLE and periodontitis may involve polymorphism of the Fcγ receptor. Moreover, SLE patients with combined Fcγ receptor risk alleles demonstrated increased periodontal disease severity compared to SLE patients without disease (Kobayashi et al., 2007; Kobayashi et al., 2010). Increased evidence has also implicated a role for periodontal disease in the potentiation of SLE pathogenesis. Reciprocally to SLE-induced exacerbation of periodontitis, periodontal pathogens may contribute to excessive immune activation of TLRs in periodontal tissues, thus contributing to systemic autoimmunity (Getts et al., 2020). SLE patients also demonstrate greater bacterial load and an increased relative abundance of oral pathogens (Jensen et al., 1999; Sete et al., 2016; Corrêa et al., 2017) compared to healthy individuals. Moreover, periodontal treatment was found to improve SLE patient responses to immunosuppressive therapy, thus supporting the concept that periodontitis may exacerbate SLE and thus prove as a potential modifiable risk factor (Fabbri et al., 2014).

### Blood

Systemic inflammatory disease states associated with periodontal disease are hypothesized to occur due to transmigration of periodontal pathogens and/or periodontitis-associated inflammatory mediators, such as IL-1, IL-6, CRP, and fibrinogen in the bloodstream (Genco and Van Dyke, 2010; Bokhari et al., 2012; D’Aiuto et al., 2013; Schenkein et al., 2020). The influence of periodontal health to systemic inflammatory processes is highlighted *via* local treatment of periodontitis that attenuates systemic inflammatory markers (Tonetti, 2009; Bokhari et al., 2012; Türer et al., 2017; D’Aiuto et al., 2018; Schenkein et al., 2020). The surface area of periodontal pockets comprises ~80-20cm (Rosan and Lamont, 2000) and may serve as a direct route for periodontopathic bacteria, their associated by-products, and periodontitis-associated immune mediators to access systemic circulation (Hajishengallis and Chavakis, 2021). Systemic perturbations, such as systemic bacteremia by periodontal pathogens, are sensed by haemopoietic stem and progenitor cells in the bone marrow *via* toll-like receptors (TLRs) and inflammatory cytokines. As a result, hematopoietic stem cells (HSCs) increase proliferation and myeloid differentiation to increase neutrophil and monocyte counts (Chavakis et al., 2019). Further differentiation of monocytic precursors leads to osteoclast precursor generation in the bone marrow and subsequent circulation in the bloodstream. In support of this, patients with periodontitis have higher counts of peripheral blood mononuclear cells that are more inclined to become RANKL-induced osteoclasts (Herrera et al., 2014). Clinical imaging *via* F-fluorodeoxyglucose positron emission tomography-computed tomography has also correlated inflammatory metabolic activities within the periodontium to increased myelopoiesis and arterial inflammation (Ishai et al., 2019). Together, such findings implicate a role for increased hematopoietic activity induced by periodontitis to inflammation at distant sites.

PCR and next-generation sequencing techniques have confirmed the presence of bacterial DNA in blood, with bacteria from the gut, reproductive tracts, skin, and oral cavity as plausible sources of blood-borne bacteria (Nikkari et al., 2001; McLaughlin et al., 2002; Amar et al., 2013; Rajendhran et al., 2013; Païssé et al., 2016; Gosiewski et al., 2017). Invasive oral procedures, such as periodontal treatment, toothbrushing, dental visits, and oral surgery are implicated in transient bacteremia (Tomás et al., 2012). Moreover, oral bacteria such as *A.* actinomycetemcomitans, *P. gingivalis, T. denticola, Prevotella intermedia, T. forsythia*, *Streptococcus mutans*, and *Streptococcus sanguinis* have been identified in cardiovascular lesions and have been commonly associated with cardiovascular diseases (Ford et al., 2005; Kozarov et al., 2005; Gaetti-Jardim et al., 2009; Leishman et al., 2010; Rafferty et al., 2011; Armingohar et al., 2014). Most of the blood-borne bacterial DNA content has largely been assigned as Proteobacteria (80-87%), followed by Actinobacteria and Bacteroidetes, suggesting that most bacterial DNA in blood is derived from the gut microbiota (Païssé et al., 2016). However, such studies have only accounted for total DNA and have not discriminated between lysed and intact bacterial cells, and as such do not reflect viable bacterial populations found in blood (Whittle et al., 2019). Emery et al. (2021) recently employed the MolYsis Complete5 system to identify only intact bacterial cells in blood from periodontally healthy and periodontally-diseased cohorts. As opposed to commonly identified *Proteobacteria* of gut origin, this study alternatively identified 43-52% of bloodborne bacteria stemming from the oral cavity, with *Proteobacteria* accounting for only low levels. *Firmicutes*, including *Streptococcus* species, accounted for nearly 65% of all bacterial sequences identified among healthy cohorts, with this number increasing only slightly in periodontitis. Subsequent groups listed in descending numbers were *Actinobacteria*, *Bacteroidetes*, *Fusobacteria*, and *Spirochaetes* that were represented in similar percentages among healthy and diseased cohorts. Only two taxa showed significantly different levels between healthy and diseased cohorts, yet both contributed to only minor components of the total microbiota: *Saccharibacteria* was not present among the diseased cohort, and *Proteobacteria, Deltaproteobacteria*, and *Myxococcales* were higher in healthy cohorts (Emery et al., 2021).

### Inflammatory Bowel Diseases

The relevance of the oral cavity to inflammatory bowel diseases (IBD) is demonstrated by extra-intestinal manifestations, such as in Crohn’s disease, that can manifest in the buccal mucosa, tongue, lips, teeth, and periodontium (Muhvić-Urek et al., 2016). Importantly, oral involvement in IBD manifests in 0.5-20% (Pittock et al., 2001; Katz et al., 2003; Zbar et al., 2012; Skrzat et al., 2017). Moreover, increased prevalence and severity of periodontitis is observed among IBD patients compared to healthy cohorts and has demonstrated the role of intestinal health to the oral cavity (Habashneh et al., 2012; Vavricka et al., 2013). Recent evidence has also suggested, however, that this relationship is bi-directional in nature, with periodontal health as an important factor to intestinal health and IBD pathogenesis. For example, in a murine model of Crohn’s disease, mice spontaneously develop periodontal inflammation, and severity of periodontitis is positively correlated to ileal inflammation (Pietropaoli et al., 2014). Moreover, oral bacteria such as *Aggregatibacter, Campylobacter, Enterobacteria, Fusobacterium*, *Gemella, Neisseria, Pastruella, Peptostreptococcus, and Streptococcus* species are enriched among mucosal tissues in IBD cohorts (Dinakaran et al., 2019; Kitamoto et al., 2020). An immunological link between periodontitis and IBD has also been hypothesized, such that immune cells activated locally in gingival tissues by periodontopathic bacteria can transmigrate to the gut and contribute to aberrant and exacerbated inflammatory processes (Morton et al., 2014). A comprehensive study by Kitamoto et al. (2020) investigated the role of periodontal disease to IBD severity *via* two potential mechanisms: 1) ectopic colonization of oral bacteria in the gut and 2) transmigration of orally primed immune cells to the gut. Mice induced with experimental colitis *via* dextran sodium sulfate treatment, together with oral ligature-induced periodontitis, experienced exacerbated gut inflammation characterized by an increased T_H_17 and T_H_1 signature. Upon examination of the oral and gut microbiomes, mice subjected to experimental periodontitis and colitis exhibited oral dysbiosis with a greater representation of Enterobacteriaceae, such as *Klebsiella* and *Enterobacter* species among the oral and gut microbiotas. Interestingly, experimental colitis alone did not result in increased abundance of Enterobacteriaceae, and moreover, experimental periodontitis alone did not enrich these species in the gut (Kitamoto et al., 2020). These findings are consistent with previous studies that identify unique microbial signatures among periodontitis-colitis patients compared to colitis patients without oral involvement, in which IBD-associated with periodontitis enriched for gram-negative rods such as *Campylobacter (* Liu et al., 2018; Graves et al., 2019). Such findings suggest a synergistic effect of periodontal disease and colitis in exacerbating inflammatory bowel disease, such that a healthy gut microbiota may resist ectopic colonization by periodontal pathogens.

The microbial-immunological axis between periodontitis and IBD was also explored: elevated IL-1β secretion is associated with both IBD and periodontitis (Park et al., 2014; Seo et al., 2015). Colitis-susceptible germ-free mice colonized with dysbiotic oral microbiotas from mice subjected to ligature-induced periodontitis displayed increased IL-1β signatures compared with germ-free mice colonized with healthy oral microbiotas. Further examination revealed that IL-1β-producing cells were primarily comprised of inflammatory macrophages. IL-1β is also identified as a significant factor in oral-pathogen mediated gut inflammation, as treatment with IL-1 receptor antagonist largely attenuated colitis in germ-free mice colonized with a periodontitis-associated oral microbiota. Moreover, the significance of oral pathobionts, but not gut pathobionts, to IL-1β secretion in the gut was confirmed, thus suggesting that IL-1β secretion in colitis may be attributed to oral pathogens. The transmigration of immune cells from periodontal tissues to the gut was also investigated. As T_H_17 and T_H_1 cells were significantly enriched in mice with both experimental periodontal disease and colitis, a possible mechanism by which such T cells transmigrate from the oral mucosa to the gut was suggested. Periodontitis induced generation of oral-pathobiont specific IL-17A^+^ T_H_17 memory T cells that accumulated in the cervical lymph nodes. Oral T memory cells isolated from the cervical lymph nodes of ligature-mice were transferred to germ-free mice colonized by oral bacteria from mice subjected to ligature-induced periodontitis. Transfer of the T memory cells elicited colitis development and oral T memory expansion in the colonic mucosa, whereas this was not observed for germ-free mice colonized *via* transfer of a healthy oral microbiota (Kitamoto et al., 2020). Collectively, this study identified microbiological and immunological connections linking periodontitis to IBD pathology: oral bacterium reactive T cells that transmigrate to the gut in existing colitis and subsequent activation of such orally primed T cells *via* ectopic colonization by oral pathogens.

### Oral Cancer

Periodontal disease has been implicated in oral squamous cell carcinoma (OSCC) potentiation *via* both microbial and immunological mechanisms. Pathologic microbial shifts within the oral microbiota characteristic of periodontitis give rise to an in increase in the relative abundance of putative periodontal pathogens, including *P. gingivalis, T. denticola*, and *T. forsythia* that appear in later stages of oral biofilm development. Increased abundance of such pathogens is correlated to periodontal disease severity (Simonson et al., 1988; Yoshida et al., 2004). Pathologic shifts in the oral microbiota have been associated with oral cancer (Whitmore and Lamont, 2014) and with specific microbial shifts associated with primary and metastatic head and neck squamous cell carcinomas (**Figure 1**) (Shin et al., 2017). While such associations between pathological shifts in the oral microbiota during periodontitis and OSCC have been suggested, the mechanisms underlying the synergistic effects of such disease states remain largely unexplored. Kamarajan et al. (Kamarajan et al., 2020) recently investigated the role of specific periodontal pathogens, including *P. gingivalis, T. forsythia*, and *F. nucleatum*, in carcinogenesis of OSCC *via* measuring the effects of such pathogens on cell migration, invasion, stemness, and tumor aggressivity, and additionally determined possible mechanisms by which such organisms promote OSCC progression. The effects of periodontal pathogens on OSCC cell migration were evaluated *via* a scratch migration assay, in which increased cell invasion was promoted by each pathogen. Tumorsphere formation of OSCC cells was additionally elevated by each pathogen. A murine floor-of-mouth model corroborated *in vitro* findings such that mice injected with pathogen-challenged OSCC cells demonstrated increased tumor burden compared to those injected with pathogen-free OSCC cells.

As integrin alpha V is central to OSCC migration, the role of each pathogen to utilize this to promote increased cell migration and stemness was also investigated to better underscore the mechanistic underpinnings of periodontal pathogens in OSCC pathogenesis. Integrin alpha V was significantly upregulated in OSCC cells upon challenge with pathogens compared to controls. Conversely, upon suppressed expression of integrin alpha V, pathogen-induced migration was attenuated in OSCC cells. As cell migration is dependent on integrin binding to the extracellular matrix, leading to recruitment of focal adhesion kinase (FAK), the role of FAK in pathogen-induced cell migration was also investigated. Challenge with *T. denticola* enhanced FAK phosphorylation in a dose-dependent manner, and alternatively suppression of FAK signaling attenuated pathogen-induced integrin alpha V expression and FAK phosphorylation. The intersection of TLR/MyD88 and integrin/FAK signaling was additionally investigated, in which suppression of MyD88 prevented phosphorylation of FAK by *T. denticola*. The mechanistic underpinnings were further explored *via* investigating the role of specific bacterial factors in OSCC. Purified lipo-oligosaccharide from *T. denticola* and LPS from *P. gingivalis* and *F. nucleatum* were able to promote OSCC migration. Interestingly, LPS derived from commensal species *V. parvula* was unable to induce the same effects (Kamarajan et al., 2020).

Inflammatory processes in the gingiva resulting from periodontitis induce environmental modifications of the periodontal tissues, such as elevated levels of reactive oxygen species, volatile sulfur compounds, acetaldehyde, lactic acid, acetic acid, butyric acid, and isocaproic acid (Karpiński, 2019) that have the potential to alter cell behavior and extracellular matrix components *via* increased host cell DNA damage. Such changes promote increased cell invasion, proliferation, and seeding of metastatic tumor cells. Periodontitis severity is associated with increased DNA damage in the buccal mucosa, reflected by increased nuclear bud formation and chromosomal instability (Borba et al., 2019). The role of periodontal disease in potentiation of OSCC was suggested in a murine model of OSCC induced by 4-nitroquinoline-1-oxide carcinogen, in which the size and number of cancerous lesions were increased upon co-infection with orally introduced periodontal pathogens, as opposed to germ-free mice that received carcinogen treatment alone (Stashenko et al., 2019). Pathogenic bacteria in periodontitis influence immune-regulatory networks, including cytokines, chemokines, and growth factors, that can attenuate and interrupt immune surveillance (Hajishengallis and Lambris, 2012). Such mechanisms not only allow pathogenic bacteria to thrive in the periodontium but may additionally encourage seeding of metastatic tumor cells and/or promote primary malignant lesions (Elebyary et al., 2021). For example, *P. gingivalis* has also been shown to disrupt immune effectiveness *via* activation of STAT3, thus leading to generation of immunosuppressive myeloid-derived suppressor cells that help to retain OSCC cell proliferation and encourage escape from immune surveillance (Arjunan et al., 2018). Moreover, the re-programming and subversion of immune cell populations, such as polymononuclear neutrophils, by periodontopathic bacteria creates a cycle of reciprocal reinforcement that perpetuates inflammation in the gingiva while encouraging outgrowth of pathogens may also contribute to OSCC development. Increased immunosuppressive IL-10 cytokines produced by polymorphonuclear neutrophils were observed among periodontitis patients and the interaction of such neutrophils with regulatory T cells stimulated with lipopolysaccharide induced the production of IL-10 (Lewkowicz et al., 2016). Elevated IL-10^+^ polymorphonuclear neutrophils were similarly characterized among OSCC patients, thus uncovering possible links by which periodontal infection may promote OSCC *via* reduced immune regulatory processes. OSCC is also associated with increased oral neutrophil counts that are correlated to poor prognosis and higher recurrence incidence (Shen et al., 2014).

### Coronavirus Disease-2019 (COVID-19)

COVID-19 is a characterized by a wide variety of symptoms, ranging from mild phenotypes such as fever, dry cough, fatigue, loss of taste and/or smell to more severe symptoms, such as dyspnea, acute respiratory distress, and multi-organ failure (Sharma et al., 2016; Yang X. et al., 2020). Although most cases are mild in nature, 14% of confirmed cases require hospitalizations and oxygen support, 5% require intensive care unit treatment, and 2% are fatal. Severe symptom development is associated with excessive levels of pro-inflammatory cytokines and systemic tissue destruction, dubbed cytokine storm syndrome (Yang Y. et al., 2020). Disease mortality is positively correlated to elevated serum pro-inflammatory mediators, including IL-6, CRP, D-dimer, and ferritin levels that demonstrate the link between hyper-inflammatory responses and disease severity (Chen et al., 2020; Ruan et al., 2020). Further, various comorbidities associated with systemic inflammation, including cardiovascular disease, obesity, and diabetes, are additional risk factors for severe COVID-19 symptoms and poor prognosis (Wu et al., 2020; Zhou et al., 2020). Periodontitis is a chronic inflammatory condition that is linked to systemic inflammatory responses and co-morbidities, as demonstrated in the previous sections. In a case control study by Marouf et al. (2021), periodontal status was evaluated alongside COVID-19 severity. Cases were characterized as patients diagnosed with COVID-19 and complications including intensive care unit (ICU) admission, ventilation, and/or death. Controls were identified as COVID-19 patients without corresponding severe complications. Periodontal status was defined as bone loss detected radiographically, with healthy patients defined as having >15% of the coronal third of root length or <2mm bitewing radiographs, and periodontally diseased patients as having bone loss >15% of the coronal third or root length or >2mm in bitewing radiographs. A total of 568 patients were included in the study and, among these, 40 experienced severe complications. >80% of patients with COVID-19 complications had periodontitis, compared to those without complications, of which 43% demonstrated some degree of periodontal disease. Of the 568 patients, 258 presented with periodontitis, and 33 of these developed COVID-19 complications. In comparison, only 7 of the 310 patients without periodontitis developed severe COVID-19 complications. After adjusting for comorbidities such as diabetes and hypertension, periodontitis maintained a significant impact on development of severe COVID-19 complications, death, ICU admission, and need for ventilation. Fatal COVID-19 outcomes were significantly associated with increased inflammatory mediators detected in blood. COVID-19 patients with periodontitis had higher white blood cell and CRP levels compared to those without periodontitis, suggesting that periodontitis contributes to COVID-19 severity *via* systemic inflammation (Marouf et al., 2021).

Several mechanisms have been hypothesized as to how periodontal disease may potentiate COVID-19 severity, including aspiration of periodontal pathogens, leading to increased expression of angiotensin-converting enzyme 2 (ACE2) and increased cytokines in the lower respiratory tract (Takahashi et al., 2021), periodontal pathogen-induced viral virulence *via* cleavage of S glycoproteins (Madapusi Balaji et al., 2020; Takahashi et al., 2021), and by way of the oral cavity acting as a viral reservoir (Badran et al., 2020; Badran et al., 2020; Botros et al., 2020; Kheur et al., 2020; Madapusi Balaji et al., 2020), in addition to increased inflammatory response pathways with systemic consequences (Sahni and Gupta, 2020). SARS-CoV-2, the causative agent in COVID-19, is an airborne coronavirus that is transmitted *via* exposure to infected droplets and aerosols *via* speaking, breathing, coughing, sneezing, and other actions involving the oral cavity (Ghinai et al., 2020; Pung et al., 2020; Hamner et al., 2020). SARS-CoV-2 utilizes *ACE2* and *TMPRSS* host receptors to enter host cells (Hoffmann et al., 2020; Zang et al., 2020). The cell types expressing such receptors vary widely throughout the body (Singh et al., 2020; Sungnak et al., 2020; Brann et al., 2020). Oral manifestations of COVID-19, including loss of taste, manifest clinically in approximately 50% of all cases. Albeit this, few studies have identified the capability of the virus to directly replicate in oral tissues, which could be greatly important to understanding the role of the oral cavity in virus transmission to other individuals, as well as to the gastrointestinal tract *via* saliva. Huang et al. (Huang et al., 2021) recently identified 34 unique cell subpopulations within the gingiva and salivary glands that harbor SARS-CoV-2 viral entry factors, in which infection was non-uniform across intra-oral sites, consistent with the heterogeneity of the oral cavity. Single-cell RNA sequencing and fluorescence *in situ* hybridization validated the expression of *ACE2* and *TMPRSS2* expression in the salivary glands and gingival mucosa. Expression was also identified among the buccal mucosa, ventral/dorsal tongue, soft palate, and palatine/lingual tonsils, in which increased suprabasal expression was observed compared to basal compartments. Salivary fractions, both acellular (from infected salivary glands) and cellular (shed from infected mucosa), were also tested and confirmed among asymptomatic and symptomatic individuals, highlighting the significance that expelled salivary droplets may contribute to spreading infection. Moreover, perceived loss of taste and smell was positively correlated with salivary levels of SARS-CoV-2 RNA (Huang et al., 2021).

## In With the New: Additional Players in Periodontal Disease Etiology

### Food for Thought: The Role of Diet in Periodontal Disease

The influence of diet on caries disease progression is well-established. It’s role in periodontal disease pathology, however, has been comparatively overlooked. Primary dietary factors associated with increased risk of periodontitis include processed carbohydrates, low fiber intake, saturated fats, and high protein consumption (**Figure 1**) (Woelber and Tennert, 2020). Micronutrient deficiencies, such as vitamin C, vitamin D, vitamin B, vitamin A, magnesium, calcium, iron, zinc, potassium, copper, manganese, and selenium deficiencies, are also associated with periodontal disease incidence and severity (Dommisch et al., 2018). Current findings relating periodontal disease incidence are largely correlative in nature and focus on clinical parameters of periodontal disease, with little focus on mechanistic underpinnings. Moreover, findings have varied among investigators. Dietary pattern analysis was employed by Alhassani et al. (2021) to investigate the association between major dietary patterns and periodontal disease incidence among participants in the Health Professionals Follow-up Study across the course of 24-years. This study consisted of 51,529 male subjects who had completed a questionnaire in 1986 (aged 40-75 years at the time) and that continued to provide dietary habit information *via* food frequency questionnaires every four years (subjects with periodontitis at baseline were not included). Across the study, 3,738 new cases of periodontitis were observed in a biennial fashion. However, a significant relationship between Western (high fat, low fiber, processed carbohydrate) or prudent (whole grains, fruits, vegetables) diets with periodontal disease incidence was not observed, but periodontitis did significantly increase with Western diet consumption among obese individuals, specifically. An 11-year follow-up study performed by Jauhiainen et al. (2020) found that, among 240 individuals, poor diets [as determined by the Baltic Sea Diet (BSD) and Recommended Finnish Diet Scores (RFD)], were associated with the development of deepened periodontal pockets among middle-aged adults. Healthy diets among the RFD and BFD included fruits, vegetables, a higher white meat and fish consumption, fibrous cereal grains (rye, oats, barley), and those with a higher ratio of unsaturated fatty acids to saturated fatty acids. Negative components included salt, sucrose, red meat consumption, and alcohol intake (Alhassani et al., 2021). Woelber et al. (2019) studied the effects of an anti-inflammatory diet in patients with gingivitis. An anti-inflammatory diet low in processed carbohydrates and animal proteins, and high in omega-3-fatty acids, vitamin C, vitamin D, antioxidants, plant nitrates, and fibers was administered to the experimental group while the control group did not change Western dietary habits. Both groups abstained from inter-dental cleaning for four weeks during the experimental period. Clinical and serological parameters as well as analysis of the subgingival microbiome following the study period suggested a significant reduction (~40%) in gingival bleeding among the experimental group compared to the control. However, a significant difference in serological inflammatory parameters (TNF-α, IL-6, high-sensitivity C-reactive protein) was not observed between the two groups. Moreover, no significant changes among the subgingival microbiota were found (Woelber et al., 2019). However, metabolic capabilities among the experimental and control microbial communities were not investigated. A large limitation of the study may be due in part to a short experimental period.

High dietary carbohydrate consumption is well-recognized in dental caries (Moye et al., 2014; Wang et al., 2019). Recent studies have identified increased carbohydrate consumption as a risk factor in periodontal disease as well, thus supporting an integrated hypothesis of caries and periodontal disease (Nyvad and Takahashi, 2020). Such findings are consistent with lower oral disease burden among societies with low agricultural integration and dietary influence (Crittenden and Schnorr, 2017; Crittenden et al., 2017), as well as the emergence of dental disease incidence at the Neolithic Revolution that is paralleled by increased consumption of cereal grains (Adler et al., 2016). Hamasaki et al. found a significant association between high carbohydrate, low fat diets and periodontal disease incidence. Using a large data set among Japanese subjects from the 2005 National Health and Nutrition survey, the Comprehensive Survey of Living Conditions, and the Survey of Dental Diseases, collectively comprising 3,043 individuals, a significant association was identified between periodontal disease incidence and total calorie intake from fats. The percentage of calories from fats was significantly lower in the group with advanced periodontal disease and a low-fat, high-carbohydrate diet was associated with periodontal disease incidence (Hamasaki et al., 2017). These findings are consistent with previous studies that have identified reduced periodontal disease incidence among subjects with higher cholesterol levels (Izumi et al., 2009) and higher intake of omega-3 fatty acids (Iwasaki et al., 2010). A study by Moreira et al. (2021) additionally found a positive association between increased sugar intake and periodontal disease among adolescents. The World Health Organization recommends limiting sugar intake to <10% of total daily energy to reduce the risk of non-communicable disease(s) (WHO, 2021). As such, the association between sugar intake >10% of daily energy and periodontal disease status in adolescents was studied. Among 2,515 aged 18-19 years having completed a food frequency questionnaire (frequency and portion size of 106 food items over 12 months) along with periodontal clinical examination (visible plaque index, bleeding on probing, periodontal probing depth, clinical attachment level among 6 teeth), 34.35% of subjects had >10% daily sugar intake, and 2.31% had sugar consumption >20% total energy intake. After adjusting for sociodemographic factors and smoking/alcohol use, >10% daily sugar intake was associated with an increased number of teeth affected by periodontal disease (*p=0.011*) (Moreira et al., 2021). Such association between increased sugar/carbohydrate intake with higher periodontal disease incidence may be explained by systemic inflammation induced by hyperglycemia and advanced glycosylation end products (Aragno and Mastrocola, 2017; Nyvad and Takahashi, 2020). Increased sugar intake may act locally within oral biofilms to drive oxidative stress and microbial dysbiosis leading to periodontitis. This hypothesis is consistent with *in vitro* findings of supra- and subgingival biofilm development, in which subgingival biofilm growth was largely dependent on prior colonization by saccharolytic supragingival species and corresponding exopolysaccharide synthesis (Thurnheer et al., 2016). Given the association between periodontal disease incidence and carbohydrate consumption, corroboration from increased oral disease burden at historical eras defined by increased cereal grain consumption, and the multi-species nature of oral subgingival biofilms, such findings warrant future investigation to better understand the systemic and/or local impacts of carbohydrate consumption in periodontal disease.

### Don’t Stress It: New Implications for Psychological Stress in Periodontal Disease

Environmental perturbations, such as diet and lifestyle, to the host additionally have the capacity to alter host-microbiome homeostasis. Sustained psychological stress has been recognized as an etiological risk factor among a variety of chronic diseases, including diabetes and rheumatoid arthritis (McCray and Agarwal, 2011; Marcovecchio and Chiarelli, 2012). Activation of the central nervous system and the hypothalamus by stress results in the release of corticotropin-releasing hormone and arginine vasopressin. This further stimulates the release of adrenocorticotropin from the pituitary gland, leading to cortisol production by the adrenal cortex. This process is collectively known as the hypothalamic-pituitary-adrenal (HPA) axis (Breivik et al., 2000). Cortisol is the primary hormone related to stress response. Notably, cortisol levels increase in saliva and serum profiles in periodontitis patients, with levels that are positively associated with disease severity, and cortisol is higher in gingival crevicular fluid of periodontitis patients (Ishisaka et al., 2008; Rai et al., 2011). Moreover, glucocorticoids (including cortisol) have been shown to downregulate immune function in the oral cavity which may compromise immune response to periodontal pathogens (Genco and Van Dyke, 2010). Recent work by Duran-Pinedo et al. (2018) identified a direct role of cortisol production to perturbations of oral biofilm samples consistent with periodontal disease severity from periodontitis patient cohorts (**Figure 1**). Dental plaque samples from patients with periodontitis were treated with cortisol *in vitro* to investigate its direct effect on the oral microbiota without possible confounds due to systemic endocrine effects. Transcriptomic analysis revealed significant shifts among the phylum Fusobacteria. Gene ontology analysis was subsequently performed to better understand the gene expression profiles of the oral microbiome in response to cortisol treatment. Among changed expression profiles, proteolysis, oligopeptide transport, iron metabolism, and flagellum assembly were enriched in the cortisol-treated group (Duran-Pinedo et al., 2018). Such gene profiles are consistent with activities of the oral microbiota in periodontal disease (Duran-Pinedo et al., 2014; Yost et al., 2015). Importantly, genes linked to the host immune response were also upregulated in the cortisol-treated group, even in the absence of host cells. Fusobacteria increased transcriptional activity more significantly compared to other phyla present. However, most of the microbial community demonstrated a shift in its profile of expression, thus demonstrating that a larger fraction of species collectively up-regulated putative virulence factors. Of these, *Streptococcus* produced the greatest changes in virulence factor up-regulation. When studying the effect of cortisol on pure cultures of organisms that were identified as more active upon initial cortisol treatment, shifts in the transcriptomic profile of both *Leptotrichia goodfellowii* and *F. nucleatum* were observed. *F. nucleatum* displayed up-regulated biological processes associated with periodontitis, including proteolysis, cobalamin biosynthesis, and iron transport, also observed for *L. goodfellowii.* Both organisms additionally displayed up-regulation of genes related to lipid A biosynthesis, DNA replication or translation, iron acquisition, and peptidase activities (Duran-Pinedo et al., 2018). Collectively, this study introduces a novel, direct role for psychological stress-related hormones to perturbations in the oral microbiota that are paralleled by transcriptional shifts observed in periodontitis *in vivo.*

Psychological stress has been shown to delay wound healing *via* suppressing the host immune response (Bosch et al., 2007; Vegas et al., 2012). As such, Zhao et al. (2012) studied the effects of psychological stress on wound healing in periodontitis. Periodontal healing is mediated by various growth factors, including basic fibroblast growth factor (bFGF), which is characterized as a significant factor to periodontal ligament regeneration (Murakami et al., 1999; Lalani et al., 2005; Katayama et al., 2006), and in normal wound healing (Gospodarowicz, 1974) *via* cell proliferation, differentiation, and angiogenesis (Shimabukuro et al., 2005). As such, the investigators tested the effect of psychological stress on downregulation of bFGF expression in periodontal wound healing. Experimental periodontitis was induced in rats *via* silk ligature placement around the second maxillary molar and ligatures were removed after 1, 2, or 4 weeks. Following ligature removal, rats were subjected to chronic unpredictable mild stress (including damp sawdust for 24 hours, food deprivation for 12 hours, light-dark cycle inversion, swimming in cold or hot water for 5 minutes, and 1 hour of restraint stress) during the healing process. Rats in the periodontitis-only and periodontitis-stress groups at baseline following ligature removal demonstrated inflammatory infiltration, significant alveolar bone loss, and clinical attachment loss. Periodontitis-only mice demonstrated spontaneous soft-tissue healing and alveolar bone remodeling in the four weeks following ligature removal. Conversely, healing processes were significantly delayed among the periodontitis-stress group, with significantly greater inflammatory infiltrate, alveolar bone loss, and attachment loss compared to the periodontitis-only group at weeks 2 and 4 (*p<0.05).* Following ligature removal, the periodontitis-only group also displayed a reduction in IL-1β and TNF-α at weeks 1, 2, and 4 compared to baseline. However, IL-1β did not significantly decrease in the periodontitis-stress group following ligature removal. TNF-α did decrease; however, levels were still higher than periodontitis-only groups. Following 4 weeks after ligature removal, bFGF expression among the periodontitis-only rats recovered, contrasting with the periodontitis-stress group in which psychological stress delayed recovery of bFGF expression (Zhao et al., 2012). As such, psychological stress not only poses direct effects to the microbiome, but moreover may perpetuate disease pathogenesis by potentiating inflammation and preventing healing in periodontal tissues.

### Say Cheese: Nisin and Nanoparticles in the Fight Against Periodontitis

The current treatment modalities for gingivitis and periodontitis include proper oral hygiene practice, and scaling and root planning, in which the treatment goal is reduced periodontal pocket depth and improved clinical attachment levels (Claffey et al., 2004; Goodson et al., 2012). Antibiotic therapy has been applied as an adjunctive treatment modality to mechanical treatment to aid in clearance of subgingival pathogens that may remain following nonsurgical treatments (Barca et al., 2015). Antibiotic treatment is not without side effects, however, such as gastrointestinal side effects, alterations of the gut microbiota, and emergence of antibiotic-resistant bacterial strains (Kapoor et al., 2012; Ramirez et al., 2020). Probiotics, defined as live cultures of microorganisms which may confer health benefits to the host when administered in adequate dosage, have gained increasing recognition as adjunct treatment modalities to non-surgical periodontal therapy (Teughels et al., 2008; Morelli and Capurso, 2012). Probiotics utilized in periodontal therapy have included tablets containing live cultures of *Lactobacillus reuteri, Lactobacillus salivarius*, probiotic drinks containing *Lactobacillus casei*, chewing gum containing *Lactobacillus reuteri*, and mouthwash containing variable doses of *Streptococcus oralis, Streptococcus uberis*, and *Streptococcus rattus* to treat varying degrees of gingival inflammation and periodontitis (Nguyen et al., 2020). Nisin, an antimicrobial peptide produced by some gram-positive bacteria such as *Lactococcus* and *Streptococcus* species, demonstrates bactericidal action against a variety of gram-positive and gram-negative bacteria (Shin et al., 2015). Today, nisin is recognized by the Food and Drug Administration as a biologically safe food preservative and is utilized as a component in processed cheese (de Arauz et al., 2009). Nisin-producing bacteria have received growing attention as potential treatment modalities in treating both oral and systemic conditions, ranging from gastrointestinal diseases to various cancers. Application of highly purified nisin to salivary-derived biofilms containing *P. gingivalis, Prevotella intermedia, A. actinomycetemcomitans, T. denticola*, and *Enterococcus faecalis* inhibited growth of pathogenic bacteria, with inhibitory action increasing in a dose-dependent manner (Shin et al., 2015).

The benefits of nisin are not only demonstrated by pathogen-killing capabilities, but moreover by its lack of harmful side effects to the host. While nisin has been shown to induce pathogen killing, high doses of nisin application were not shown to induce toxic effects to oral human cell viability and proliferation (Shin et al., 2015). An interesting finding also rests upon the seemingly selective-killing capabilities of nisin. For example, the probiotic nisin-producing *L. lactis* as well as purified nisin, can decrease levels of periodontal pathogens while retaining commensal species upon application to oral biofilms spiked with periodontal pathogens (Radaic et al., 2020). Radaic et al. (2020) identified that treatment with either nisin-producing *L. lactis* or treatment with purified nisin was able to inhibit pathogenic oral biofilm growth *in vitro*, in which nisin was able to significantly to both inhibit and disrupt biofilm formation, structure, and viability. 16S rRNA sequencing of biofilms spiked with periodontal pathogens and subjected to probiotic treatment demonstrated that nisin-producing *L. lactis* was able to recover microbial diversity indices to control levels, suggesting that nisin can selectively target periodontal pathogens. At the species level, nisin-producing *L. lactis* and purified nisin treatment successfully suppressed the growth of pathogens, including *T. forsythia* and *F. nucleatum*, while increasing the proportion of commensal species such as *Neisseria flava* and *L. lactis (* Radaic et al., 2020). Such findings of nisin related to a decrease in periodontal pathogens are reflected by improved clinical parameters of periodontal disease progression, such as the ability for nisin-producing *L. lactis* to inhibit alveolar bone loss in a murine model of periodontal disease (Nguyen et al., 2020). The benefits of nisin have been shown to extend beyond periodontal disease. Kamarajan et al. (2020) identified that, in a murine model of OSCC, nisin was able to reduce oral tumorigenesis and increase the lifespan of tumor-bearing mice. Moreover, oral tumorigenesis potentiated by periodontal pathogens in mice was abrogated by nisin application, in which pathogen-enhanced cancer cell migration, invasion, tumorsphere formation, and oral tumorigenesis *in vivo* were decreased following nisin treatment. Studies of this underlying mechanism revealed that nisin was able to inhibit *T. denticola* mediated oral carcinogenesis *via* downregulation of integrin alpha V expression and FAK phosphorylation (Kamarajan et al., 2020).

While bacteriocins have emerged as novel adjunct therapies to periodontitis, drawbacks of this approach are also noted. Bacteriocin resistance by Gram-positive bacteria, *via* cell wall modifications, modification of membrane lipid phospholipids, enzymatic inactivation of bacteriocins (Zhou et al., 2014), resistance by the outer membrane of Gram-negative bacteria, and bacteriocin sensitivity to proteases such as proteinase K and pepsin (Prudêncio et al., 2015; Ansari et al., 2018), compromise the efficacy of bacteriocin-related therapeutics. Nanoscale drug delivery systems (Nano-DDS) can aid in bacteriocin delivery *via* three primary mechanisms, including 1) improvement of pharmo-kinetics, such as alteration of solubility, charge, and stability that collectively may increase the shelf-life, bioavailability, and bactericidal half-life. 2) Nano-DDS can also improve bacteriocin efficacy against bacterial resistance by protecting them from degradative enzymes produced by resistant bacteria (Zhang et al., 2010; Arthur et al., 2014; Fahim et al., 2016), and by allowing them to overcome microbial resistance mechanisms, such as by disrupting bacterial cell membranes and cell walls damaging bacterial proton-efflux pumps or inducing oxidative stress in the bacteria (Baptista et al., 2018). 3) Nano-DDS can also directly deliver bacteriocins to diseased tissues *via* extracellular or intracellular mechanisms (Anselmo and Mitragotri, 2016). Growing attention has been applied to nisin-based bacteriocin therapies in periodontitis. However, nisin use is limited by factors including structural instability and the emergence of bacteria that are tolerant and/or resistant to its action (Salmaso et al., 2004; Zhou et al., 2014; Randall et al., 2018). For example, nisin-resistance gene/protein (NSR) inactivates nisin *via* cleavage of a peptide bone between lantionine-28 and serine-29, thus resulting in a truncated nisin with decreased membrane affinity, pore formation ability, and 100-fold decrease in bactericidal capabilities (Khosa et al., 2013; Field et al., 2015). Nisin is also sensitive to environmental factors such as pH and temperature (Zhou et al., 2014). Nano-DDS has been applied to increase nisin efficacy *via* protection from enzymatic degradation, environmental factors, and decreasing bacterial resistance. For example, studies have noted increased antimicrobial effectiveness of nisin when combined with Nano-DDS, implicating that Nano-DDS allows direct delivery of nisin to the side of action (Zhou et al., 2014). Moreover, Nano-DDS application to nisin resulted in sustained release of the bacteriocin, thus increasing long-term efficacy and action. For example, the nisin/Nano-DDS system remained active for up to 50 days after inoculation, compared to only 7 days for free nisin alone. As such, Nano-DDS systems may serve as a drug reservoir (Fahim et al., 2016), in which the entrapped nisin release is slowly sustained over time *via* diffusion, bacterial degradation, and polymer erosion (Chopra et al., 2014; Alishahi, 2014; de Abreu et al., 2016). Preliminary findings by Radaic et al. 2020 have identified enhanced bacteriocin efficacy by nisin/Nano-DSS to disrupting oral biofilms, inhibiting periodontal pathogen growth, and reducing oral cancer cell viability. As such, Nano-DDS systems in combination with bacteriocins may present as novel treatment modalities to periodontal pathogen clearance in periodontitis.

Generation of reactive oxygen species (ROS) by periodontal immune cells in response to insult by bacterial pathogens contributes to periodontal disease pathogenesis by inducing oxidative damage, thus interfering with cell cycle progression, and inducing tissue damage (Hirschfeld et al., 2017; Kanzaki et al., 2017; Liu et al., 2017). Bao et al. (2018) recently investigated the role of polydopamine as an antioxidant defense platform for ROS removal in oxygen-stress induced periodontal disease using nanoparticles. Murine models of periodontitis suggested that polydopamine nanoparticles (PDA NP) successfully remove ROS and decrease periodontal inflammation. Moreover, little capacity for long-term toxicity was noted in agreeance with high biocompatibility and biodegradation of polydopamine. The capability of PDA NP to scavenge hydroxyl radicals and superoxide radicals was investigated, in which hydroxide radical removal was measured by the presence of fluorescent 2-hydroxyterephtalic acid. In the presence of PDA NP, a decrease in fluorescence was noted, suggesting that PDA NP can remove hydroxyl radicals in a concentration-dependent manner. At a PDA NP concentration of 0.125 mg/mL, nearly all hydroxyl radicals were removed. Superoxide radical scavenging was measured by the inhibition ratio of photoreduction of NBT, in which strong absorbance signals were presented with increasing superoxide radicals. When PDA NPs were added to the system, the absorption spectra significantly decreased, in which superoxide radicals were completely removed from the system when PDA NP was administered with a concentration of 0.1mg/mL. The ability of PDA NPs to remove intracellular ROS was also investigated, in which Cu2+-modified PDA NPs were used as probes to determine uptake kinetics of HGE cells. Uptake efficiency was dependent on incubation period length and was above 50% at 12 hours. Moreover, generation of intracellular ROS was reduced by 80% with PDA NP treatment at a coincubation concentration of 0.1mg/mL (Bao et al., 2018). Collectively, this data demonstrates novel implications for nanoparticle systems to not only facilitate bacteriocin delivery and bacterial killing, but additionally to attenuate inflammation and promote recovery from periodontal disease.

## Closing Remarks

Supra- and subgingival biofilm dysbiosis paired with sustained inflammation in the gingiva are cornerstones in periodontal disease onset and progression (Hajishengallis and Lambris, 2012). Putative periodontal pathogens are critical to eliciting disease and prompting host inflammation while continuing to perpetuate disease *via* immune subversion and manipulation of tissues (Maekawa et al., 2014; Hajishengallis, 2015; Malone et al., 2021). Various models have emerged that additionally highlight the critical role of oral commensal and pathobionts in promoting periodontal disease *via* mediating intricate interactions with keystone pathogens, such as *P. gingivalis, T. forsythia, A. actinomycetemcomitans*, and *T. forsythia (* Marsh, 1994; Socransky et al., 1998; Hajishengallis et al., 2011; Suzuki et al., 2013). Additionally, novel recognition of non-bacterial microbes, including various viruses in phages comprising the oral microbiota, to disease progression is becoming an area of interest (Pride et al., 2012; Gao et al., 2020). Observations have demonstrated that the oral virome is significantly changed in periodontal disease (Gao et al., 2020), thus suggesting potential mechanisms by which viral species may manipulate both bacterial and host processes in disease progression. The factors affecting microbial dysbiosis and periodontal inflammation has extended beyond the sole absence of oral hygiene to now include host environmental factors, such as psychological stress and diet (Rai et al., 2011; Duran-Pinedo et al., 2018; Woelber et al., 2019; Woelber and Tennert, 2020; Aral et al., 2020). Psychological stress and poor diet may promote periodontal disease *via* encouraging host inflammation. However, the stress hormone cortisol was shown to directly affect microbial dysbiosis and pathogen outgrowth *in vitro*, thus demonstrating a direct role for stress in affecting microbial communities (Duran-Pinedo et al., 2018). Novel concepts in periodontal immunity have introduced a role of cell senescence in promoting the inflammatory process, as well identifying unique immune responses in the gingiva that are distinct from other barrier sites (Dutzan et al., 2017; Dutzan et al., 2018; Aquino-Martinez et al., 2020; Aquino-Martinez et al., 2020). The presence of additional chronic inflammatory diseases, such as IBD, cardiovascular diseases, and autoimmune disorders have additionally been recognized as co-morbidities that may promote periodontal disease pathogenesis (Hajishengallis and Chavakis, 2021). The oral manifestation of distant pathologies has been recognized extensively and is especially highlighted in the clinical manifestation of various IBDs (Lankarani et al., 2013; Benli et al., 2021). However, a novel role of oral pathologies and disease to promoting systemic inflammatory conditions has shed light on a new concept, that being the reciprocated systemic manifestation of oral disease. This is especially emphasized by recent findings related to connections between periodontal disease and Alzheimer’s disease, in which periodontal pathogens have been identified among brain tissue of autopsy specimens (Chen et al., 2017; Dominy et al., 2019). In line with this, growing evidence supports the role of periodontal disease in potentiating COVID-19 severity and outcomes, as in the case of other chronic inflammatory diseases (Botros et al., 2020; Sahni and Gupta, 2020; Huang et al., 2021). New discoveries related to disease etiology are complemented by novel therapeutic modalities in addition to traditional non-surgical and antibiotic treatments. Probiotics, such as nisin-producing *L. lactis* not only disrupt pathogen outgrowth, but do not adversely affect commensal microbial species (Radaic et al., 2020). Purified nisin additionally has this effect in periodontal disease-associated biofilms, and moreover does not illicit harmful effects to host cells (Shin et al., 2015; Radaic et al., 2020). The integration of nanoparticle drug delivery systems moreover enhances the effect of pre-biotic nisin administration such that diseased tissues are directly targeted while negating potential and inevitable pitfalls (Bao et al., 2018; Radaic et al., 2020). Altogether, these findings excitedly integrate novel concepts into existing models of periodontal disease pathogenesis, further emphasize the role of oral health to systemic health, and continue to advance our knowledge of disease, corresponded by growing ideas for novel treatments.

## Author Contributions

LMS, MB, and YLK contributed to conception and format of the review. LMS and MB organized the information. LMS and MB wrote the first draft of the manuscript. All authors contributed to manuscript revision, read, and approved the submitted version.

## Funding

This work was supported by funding from the NIH grants R01 NIH R01DE025225 to YLK, NIH F30DE031182 awarded to LMS.

## Conflict of Interest

The authors declare that the research was conducted in the absence of any commercial or financial relationships that could be construed as a potential conflict of interest.

## Publisher’s Note

All claims expressed in this article are solely those of the authors and do not necessarily represent those of their affiliated organizations, or those of the publisher, the editors and the reviewers. Any product that may be evaluated in this article, or claim that may be made by its manufacturer, is not guaranteed or endorsed by the publisher.
